# Time‐efficient measurement of subtle blood–brain barrier leakage using a T_1_ mapping MRI protocol at 7 T

**DOI:** 10.1002/mrm.28629

**Published:** 2020-12-08

**Authors:** Marieke van den Kerkhof, Paulien H. M. Voorter, Lisanne P. W. Canjels, Joost J. A. de Jong, Robert J. van Oostenbrugge, Abraham A. Kroon, Jacobus F. A. Jansen, Walter H. Backes

**Affiliations:** ^1^ Department of Radiology & Nuclear Medicine Maastricht University Medical Center Maastricht the Netherlands; ^2^ School for Mental Health and Neuroscience Maastricht University Maastricht the Netherlands; ^3^ Department of Electrical Engineering Eindhoven University of Technology Eindhoven the Netherlands; ^4^ Department of Neurology Maastricht University Medical Center Maastricht the Netherlands; ^5^ Cardiovascular Research Institute Maastricht, Maastricht University Maastricht the Netherlands; ^6^ Department of Internal Medicine Maastricht University Medical Center Maastricht the Netherlands

**Keywords:** 7T, BBB leakage, DCE‐MRI, T_1_ mapping

## Abstract

**Purpose:**

Blood–brain barrier (BBB) disruption is commonly measured with DCE‐MRI using continuous dynamic scanning. For precise measurement of subtle BBB leakage, a long acquisition time (>20 minutes) is required. As extravasation of the contrast agent is slow, discrete sampling at strategic time points might be beneficial, and gains scan time for additional sequences. Here, we aimed to explore the feasibility of a sparsely sampled MRI protocol at 7 T.

**Methods:**

The scan protocol consisted of a precontrast quantitative T_1_ measurement, using an MP2RAGE sequence, and after contrast agent injection, a fast‐sampling dynamic gradient‐echo perfusion scan and two postcontrast quantitative T_1_ measurements were applied. Simulations were conducted to determine the optimal postcontrast sampling time points for measuring subtle BBB leakage. The graphical Patlak approach was used to quantify the leakage rate (*K_i_*) and blood plasma volume (*v_p_*) of normal‐appearing white and gray matter.

**Results:**

The simulations showed that two postcontrast T_1_ maps are sufficient to detect subtle leakage, and most sensitive when the last T_1_ map is acquired late, approximately 30 minutes, after contrast agent administration. The in vivo measurements found *K_i_* and *v_p_* values in agreement with other studies, and significantly higher values in gray matter compared with white matter (both *p* = .04).

**Conclusion:**

The sparsely sampled protocol was demonstrated to be sensitive to quantify subtle BBB leakage, despite using only three T_1_ maps. Due to the time‐efficiency of this method, it will become more feasible to incorporate BBB leakage measurements in clinical research MRI protocols.

## 1 INTRODUCTION

An intact healthy blood–brain barrier (BBB) maintains the brain homeostasis by preventing neurotoxins from entering the brain tissue, ensuring delivery of nutrients and removal of waste products.^1^ Disruption of the BBB leads to an increased permeability, allowing unwanted biomolecules to leak from the cerebral circulation into the brain parenchyma. Previous studies have shown that disruption of the BBB is associated with developing cerebral diseases, such as Alzheimer’s disease, multiple sclerosis, stroke, and dementia.^1^, ^2^

The commonly used in vivo imaging method to quantify BBB permeability is DCE‐MRI.^3^, ^4^ During DCE‐MRI, a contrast agent (CA) is injected intravenously, while continuously acquiring T_1_‐weighted images with a high temporal resolution.^5^ The CA leakage, resulting from a disrupted BBB, can be detected on the T_1_‐weighted images, as CA molecules lower the longitudinal relaxation time (T_1_) of the brain tissue and therefore affect the MR signal intensity. These changes in signal intensity can be converted to CA concentrations, and these concentrations can be used as input for a suitable pharmacokinetic model to calculate the leakage rate. However, despite becoming widely applied at various clinical research sites, measurements of subtle BBB leakage are performed with a large variation in DCE‐ MRI acquisition settings, such as temporal and spatial resolution, and total acquisition time, which complicates the comparison among the leakage rate values reported.^4^, ^6^ Recommendations for a more standardized DCE‐ MRI protocol have been published; however, further improvements need to be made regarding the time‐efficiency of subtle BBB leakage measurements.^4^

For measurements of subtle leakage rates, with slow extravasation of CA, it is recommended to use a long acquisition time (> 20 minutes), to ensure that a sufficient amount of CA has leaked into the extravascular space of the brain parenchyma.^4^, ^6^, ^7^, ^8^ Therefore, the signal intensity changes in brain tissue are relatively small between two consecutive T_1_‐weighted images, which are typically acquired within 30‐60 seconds after each other. Considering these slow signal intensity changes, it might be sufficient to sample only a few data points over a longer time interval, with a higher SNR, rather than a continuous dynamic measurement, in which each time point has a lower SNR and spatial resolution, as performed in conventional DCE‐MRI. Moreover, measuring at only two time points after contrast injection could gain scanning time, which can be used for additional sequences, such as contrast‐enhanced MRA, fluid‐attenuated inversion recovery (FLAIR), DTI, or SWI.^9^, ^10^, ^11^

Another challenge for comparing leakage rates measured with different DCE‐protocols on various scanners is the machine‐dependent implementation of pulse sequences. However, using quantitative T_1_ measurements for the calculation of the CA concentration, rather than signal intensity changes, the influence of interscanner inconsistencies, coil inhomogeneities, scanner drift, and acquisition parameter details on BBB leakage‐rate measurements should be reduced.^4^, ^12^, ^13^, ^14^

In this proof‐of‐principle study, we explore the feasibility of a sparsely time‐sampled DCE‐protocol, consisting of a limited number of quantitative T_1_ maps, for the detection and quantification of subtle BBB leakage at 7 T MRI. Computer simulations were performed to determine the most optimal sampling time points of the postcontrast T_1_ measurements. In vivo measurements were performed in subjects without neurological conditions to measure BBB leakage rates (*K_i_*) and blood plasma volume fractions (*v_p_*) in white matter (WM) and gray matter (GM), and to demonstrate the possibilities and settings of the time points of the sparsely time‐sampled in vivo MRI protocol.

## 2 METHODS

### 2.1 Computer simulations

Computational simulations were performed in *MATLAB* (R2016a; MathWorks, Natick, MA) to investigate two properties of the sparsely time‐sampled protocol*:* (1) minimum number of T_1_ maps required for leakage detection and (2) the optimal time interval between two postcontrast T_1_ maps.

In each simulation, realistic brain tissue concentration curves over time were generated by the extended Tofts model.^15^ This model was used because it allows CA transfer back into the vascular compartment, which may become relevant for long scan periods. Based on our in vivo results, the “true” leakage rate (*K_i,_*_true_) was chosen to be 5.0 × 10^−4^ min^−1^ for WM and 8.0 × 10^−4^ min^−1^ for GM, and *v_p_* was chosen to be 0.036 and 0.049, respectively. Furthermore, to simulate an impaired BBB, 20% higher *K_i_* values were used as input, which was approximately the mean effect size reported by Montagne et al.^16^ The vascular input function, based on the concentration in the superior sagittal sinus, and the volume fraction of the extravascular extracellular space (*v_e_* = 0.05) were given as additional input to the model.^7^ Subsequently, normally distributed noise of 2.0 × 10^−3^ mM was added to the brain tissue concentration curves to mimic noise that was present in our in vivo data.

For each set of input parameters, the simulations were repeated with 250,000 runs. The graphical Patlak analysis was used to determine *K_i_* [min^−1^] and *v_p_*, as this method is considered most suitable for quantifying subtle BBB leakages.^7^, ^17^, ^18^ In the Patlak plot, the slope of the regression line represents *K_i_*, and the intercept with the vertical axis resembles *v_p_*.

Moreover, the simulations were used to evaluate the precision and accuracy of the *K_i_* obtained with the sparsely time‐sampled protocol. The SD of *K_i,_*_calculated_, representing the SD over all simulations, was calculated for evaluation of the precision. As a measure of accuracy, the percentage difference, defined as (*K_i,_*_calculated_/*K_i,_*_true_ − 1*)* × 100%, was used.

1. Minimum number of T_1_ maps required for leakage detection

The effect on the precision and accuracy of *K_i_* measurements when acquiring different numbers of postcontrast T_1_ maps, with a minimum of two postcontrast T_1_ maps, was investigated by performing simulations. The time points of the first and the last T_1_ map were set to 4 minutes and 20 seconds, and 30 minutes after the start of CA injection, respectively. This final time point considered the benefits of scanning as late as possible against clinical feasibility. The number of additional T_1_ maps that were acquired in the free time slot was varied from 0 to 5, where 5 corresponds to continuously acquiring T_1_ maps (i.e. no time gap). The time points of the additional T_1_ maps were equally distributed between the first and the last T_1_ map.

- Optimal time interval between two postcontrast T_1_ maps

The time point of the second postcontrast T_1_ map was varied in these simulations to obtain the most suitable timing of this scan based on *K_i_* accuracy and precision. This time point was varied from 9 minutes after CA injection to 39 minutes, in steps of 2 minutes. In these simulations, only two postcontrast T_1_ maps were used.

### 2.2 In vivo measurements

#### 2.2.1 Study population

Five subjects without neurological conditions (mean age 62 years; range 45‐74 years; 3 males) were included in this study. Exclusion criteria were contraindications for undergoing MRI or gadolinium administration (estimated glomerular filtration rate <30 mL/min, allergy), diabetes mellitus, history of cardiovascular disease, and body mass index > 32 kg/m^2^.

All subjects provided written informed consent before participating in the study. The study was approved by the local Medical Ethical Committee of Maastricht University Medical Center.

#### 2.2.2 Magnetic resonance imaging acquisition

**FIGURE 1:**
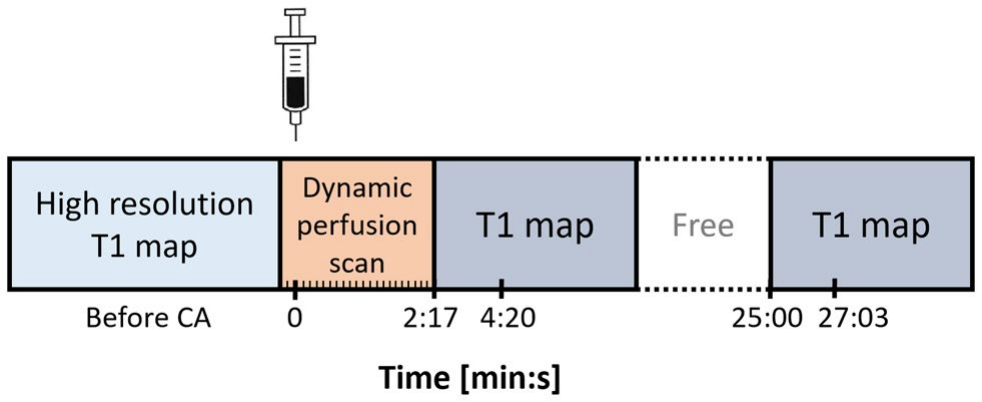
The approximate time points of the sparsely sampled protocol applied for the in vivo measurements. The time point of the last T_1_ map is scheduled to be acquired approximately 25 minutes after contrast agent injection. The second time point for each postcontrast T_1_ map represents the time point at which the center of the k‐space is acquired

**FIGURE 2:**
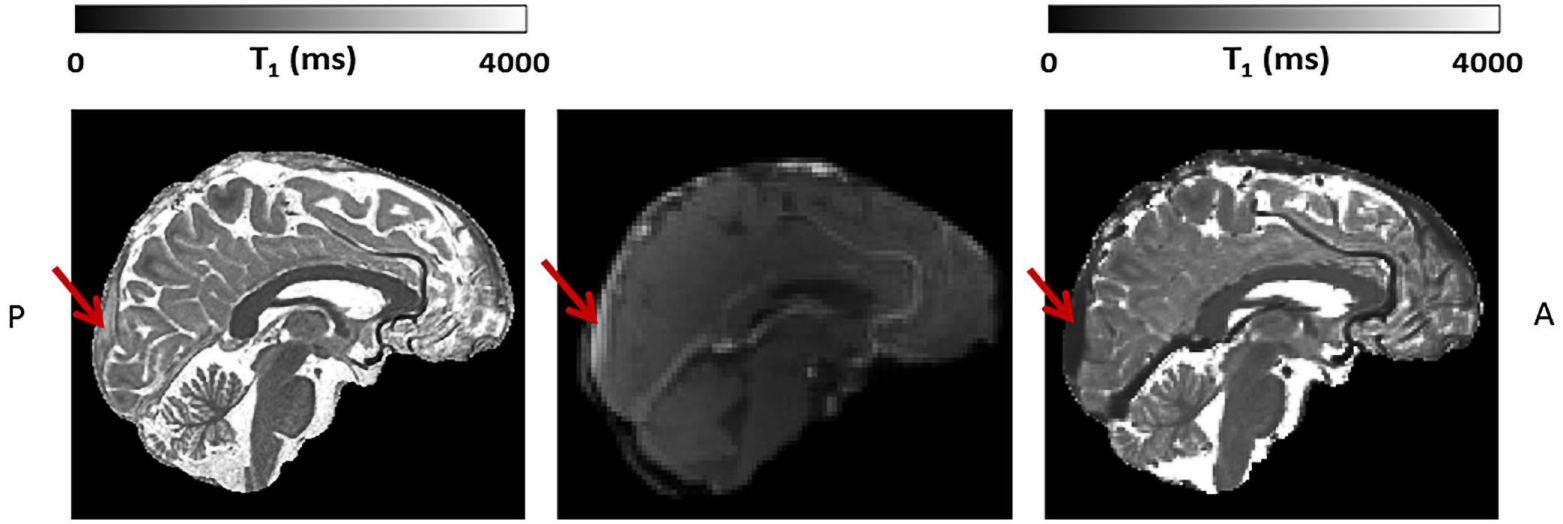
Example slice of the high‐resolution precontrast T_1_ map (left), one dynamic perfusion slice obtained after contrast agent (CA) administration (middle), and the postcontrast T_1_ map (right) (all images skull‐stripped). The FOV of the dynamic perfusion scan captures only a small midsagittal part of the brain, containing the superior sagittal sinus (SSS), indicated with the red arrow. Note the lower T_1_ relaxation time values in the SSS, seen as a high signal intensity after CA administration on the dynamic perfusion scan (middle), and as a lower signal intensity on the T_1_ map (right)

All subjects were scanned with a 7 T MRI system (Magnetom; Siemens Healthineers, Erlangen, Germany) using a 32‐element channel phased‐array head coil. For improvement of $B_{1}^{+}$ field homogeneity across the brain, dielectric pads were placed on both sides of the neck, proximal to the temporal lobes.^19^ The DCE‐protocol consisted of a precontrast T_1_ map, a 3D fast gradient‐echo T_1_‐weighted (volumetric interpolated brain examination) sequence, capturing only a small midsagittal part of the brain, to sufficiently sample the CA bolus peak, and two postcontrast T_1_ maps (see Figures 1 and 2). First, the precontrast T_1_ map was acquired using an MP2RAGE sequence.^20^ Subsequently, the dynamic perfusion scan was applied. After the first three volumes were acquired, the contrast agent (1.0 M gadobutrol, 3 mL for each subject) was injected with an infusion rate of 0.3 mL/s followed by a saline flush (20 mL). Due to the slow infusion rate, the bolus peak appeared approximately at the 30th dynamic scan of the 90 volumes. The first postcontrast T_1_ map was acquired immediately after the dynamic perfusion scan series, and the last T_1_ map was acquired approximately 25 minutes after the start of CA injection. The details of these sequences are listed in Table 1. The precontrast T_1_ map was optimized for optimal delineation of WM and GM, whereas the acquisition parameters of the postcontrast T_1_ maps enabled an accurate measurement of the expected postcontrast T_1_ values of brain tissue and blood.

**TABLE 1:** Scan parameters of the two MP2RAGE sequences, which yielded the T_1_ maps, and the sequence parameters of the dynamic perfusion sequence

	Precontrast MP2RAGE	Postcontrast MP2RAGE	Dynamic perfusion scan
TR/TE (ms)	5000/2.47	4000/2.32	3.7/1.3
TI_1_/TI_2_ (ms)	900/2750	900/2200	N/A
α_1_/α_2_ (degrees)	5/3	4/5	6.5
Voxel size (mm^3^)	0.7 × 0.7 × 0.7	1.2 × 1.2 × 1.2	2.0 × 2.0 × 2.0
FOV (mm^3^)	168 × 224 × 224	192 × 230 × 230	192 × 192 × 32
Total acquisition time (minutes)	8:00	4:16	2:47
GRAPPA factor	3	3	3
Partial Fourier	6/8	6/8	6/8
Number of volumes	1	1	90
Time interval (seconds)	N/A	N/A	1.86

To facilitate optimal segmentation of WM and GM, a T_2_‐weighted FLAIR sequence (TR/TE/TI = 8000/303/2330 ms; FOV = 192 × 192 × 176 mm^3^; voxel size = 1 × 1 × 1 mm^3^; acquisition time = 6:59 minutes) was applied in between the two postcontrast T_1_ maps.

#### 2.2.3 Data analysis

All images were spatially registered to the first postcontrast T_1_ map (FMRIB’s linear image registration tool, version 6.0) to correct for head displacements.^21^ The precontrast T_1_ map and the FLAIR images were used as input for automated brain tissue segmentation (FreeSurfer version 6.0.5).^22^ The output was visually inspected and manually corrected when required.

To obtain the vascular input function, the concentration in the blood was derived from a region of interest with a diameter of approximately 4 mm, which was manually drawn in the superior sagittal sinus. The CA concentration of blood was corrected for the hematocrit level (Hct = 0.45) to obtain the concentration in blood plasma (C_p_(t)).

For calculation of the leakage rate maps, pharmacokinetic analysis was performed using the graphical Patlak approach. The tissue concentrations were obtained directly from the T_1_ maps, as the change in the longitudinal relaxation rate (ΔR_1_) is linearly related to the CA concentration.^14^ The relaxivity constant was set to 4.2 mM^−1^s^−1^ for 7 T.^23^ The blood plasma curve was fitted by a mono‐exponential curve through the last data point of the concentration time curve of the dynamic perfusion scan, and the two MP2RAGE data points.

The *K_i_* and *v_p_* were calculated for all brain voxels, derived from the Patlak plot; and for WM and GM regions the average values were calculated. To correct for outliers, all voxel *K_i_* and *v_p_* values in these regions of interest within the 95% confidence interval were included in the analysis.

#### 2.2.4 Statistics

The *K_i_* and *v_p_* values were compared between WM and GM using a nonparametric two‐sided paired Wilcoxon signed‐rank test, with a significance level α of 0.05.

## 3 RESULTS

### 3.1 Computer simulations

1. Minimum number of T_1_ maps required for leakage detection

**FIGURE 3:**
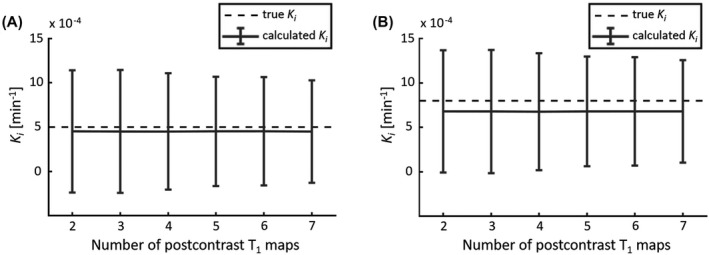
The mean and SD (error bars) of the calculated *K*_*i*_ obtained with simulating a varying number of postcontrast T_1_ maps. The dashed line represents the mean “true” *K*_*i*_ value of white matter (WM) (A) and gray matter (GM) (B). Note the relatively large SD, which is due to the high noise level, and the slightly decreasing SD when more T_1_ maps are acquired

Figure 3 shows the decrease in SD of the calculated *K_i_* for an increasing number of postcontrast T_1_ maps for WM (Figure 3A) and GM (Figure 3B). For these two input values of *K_i_*, and impaired WM and GM, the measurement precision, which is inversely proportional to the SD of *K_i_*, decreased by a factor 1.2 when two (SD[*K_i_*] = 6.9 ×10^−4^ min^−1^) instead of seven (SD[*K_i_*] = 5.5 × 10^−4^ min^−1^) postcontrast T_1_ maps were acquired, the latter representing continuous scanning. The difference in the accuracy of the *K_i_* calculated for seven postcontrast T_1_ maps versus two postcontrast T_1_ maps was less than 0.5%. It could be observed that for an increased leakage rate, the accuracy decreased.

- Optimal time interval between two postcontrast T_1_ maps

**FIGURE 4:**
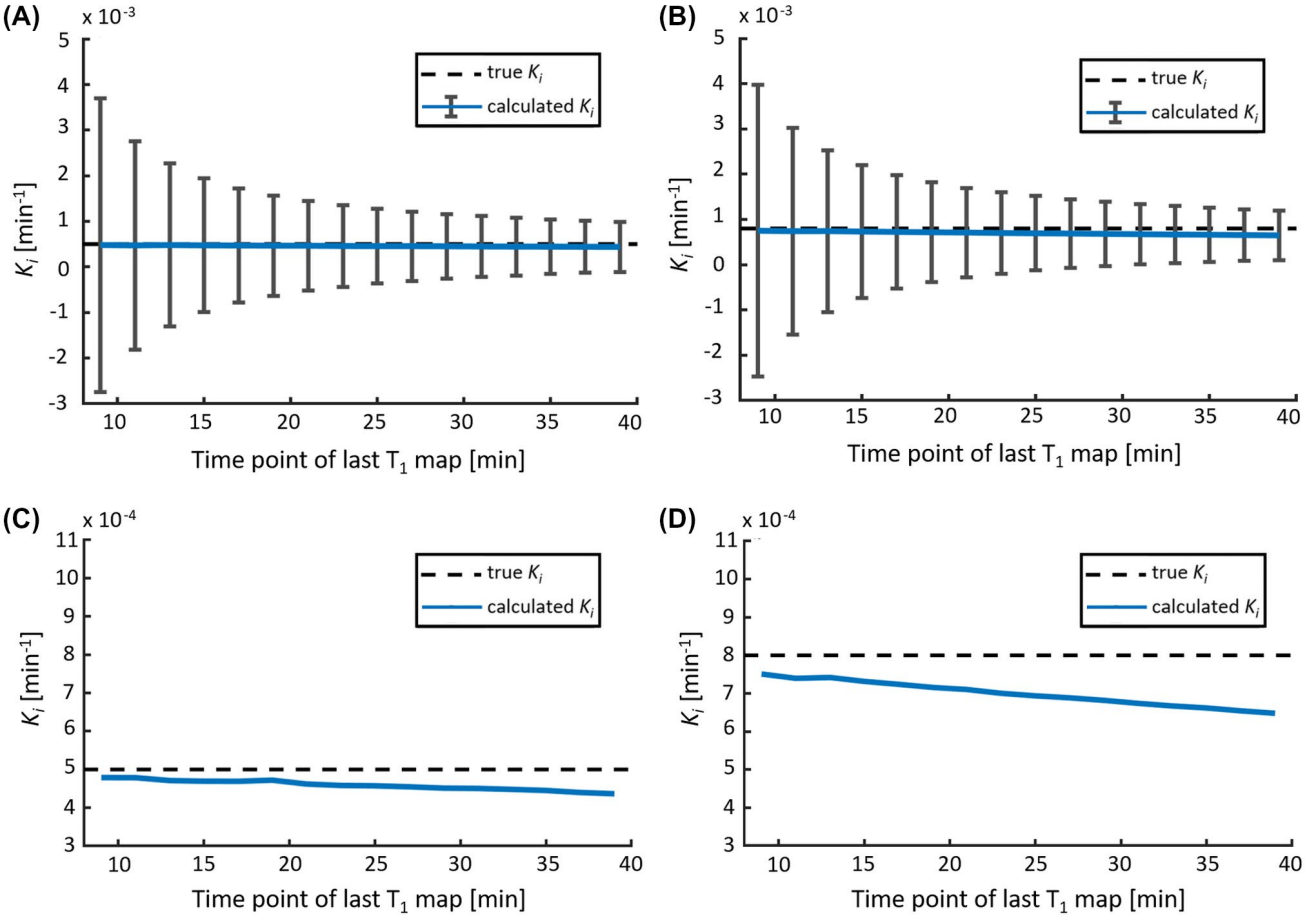
The calculated leakage rate *K*_*i*_ as a function of the time point of the second postcontrast T_1_ map (varying from 9 to 39 minutes after CA administration), which is the last T_1_ map in this simulation. The mean calculated *K*_*i*_ (blue line) with the SD (error bars) for WM (A) and GM (B). Note that the SD decreases when the last T_1_ map is acquired at a later time point after CA administration. C,D, The mean calculated *K*_*i*_ in comparison with the “true” *K*_*i*_ (dashed line), as a function of the time point of the last T_1_ map in more detail for WM (C) and GM (D). Note that the accuracy (negative bias) of *K*_*i*_ slightly decreases for a later T_1_ map, and the accuracy is lower for higher “true” *K*_*i*_ values

The simulations showed a higher precision in *K_i_* when the last T_1_ map is acquired at a later time point (Figure 4A,B). This figure also shows that acquiring the last T_1_ map later than 25 minutes after CA injection yielded a lower SD (< 8.2 × 10^−4^ min^−1^) compared with earlier time points (e.g. 9 minutes after CA injection [SD = 3.2 × 10^−3^ min^−1^]). The SD still decreases for later time points; however, the results obtained at 25 minutes (8.2 × 10^−4^ min^−1^) and 39 minutes (5.5 × 10^−4^ min^−1^) show a small difference. From Figure 4C,D it can be noted that a negative bias for *K_i_* was found for all time points, when comparing the mean calculated *K_i_* to the true *K_i_*. Due to the incorporated backflux, this effect increased for T_1_ maps acquired at later time points. Comparison of the underestimation of the calculated *K_i_* for WM from the sampling time points at 25 versus 9 minutes after CA injection showed that the percentage difference was −8.5% versus −4.2%, respectively. For increasing *K_i_*, an increasing underestimation of the calculated *K_i_* was observed. For GM, the percentage difference was −13.3% and −6.1% for 25 minutes and 9 minutes, respectively.

### 3.2 In vivo measurements

**FIGURE 5:**
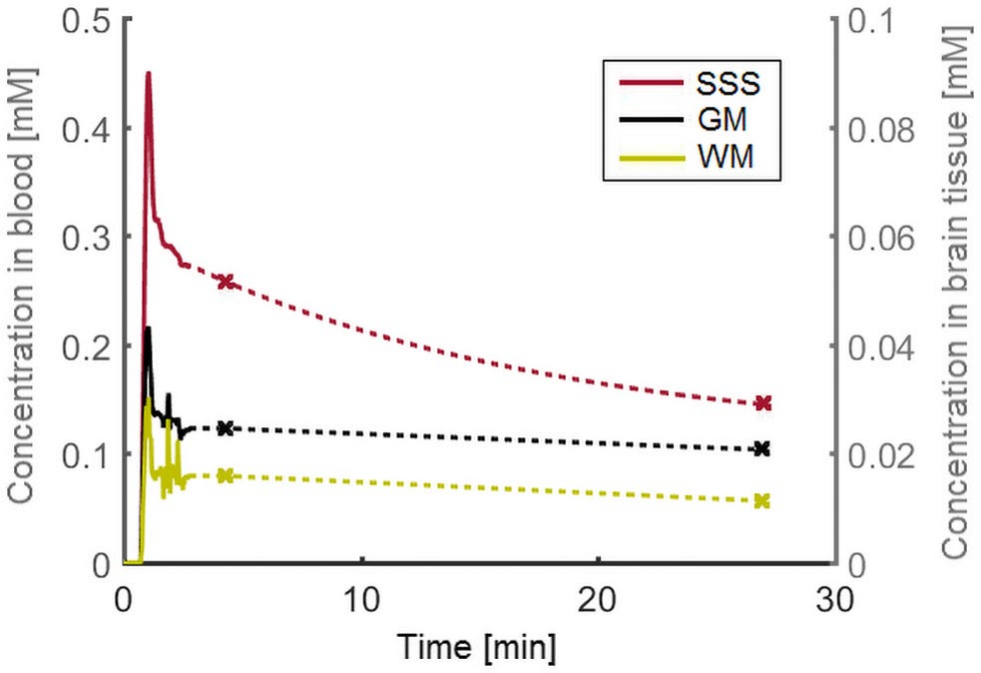
Example of the measured concentration curves for venous blood (SSS), WM, and GM. The solid line represents the sample points obtained with the dynamic perfusion sequence. The two data points derived from the postcontrast T_1_ maps are indicated with a marker. Note the different concentration axes for blood (left) and tissue (right)

**FIGURE 6:**
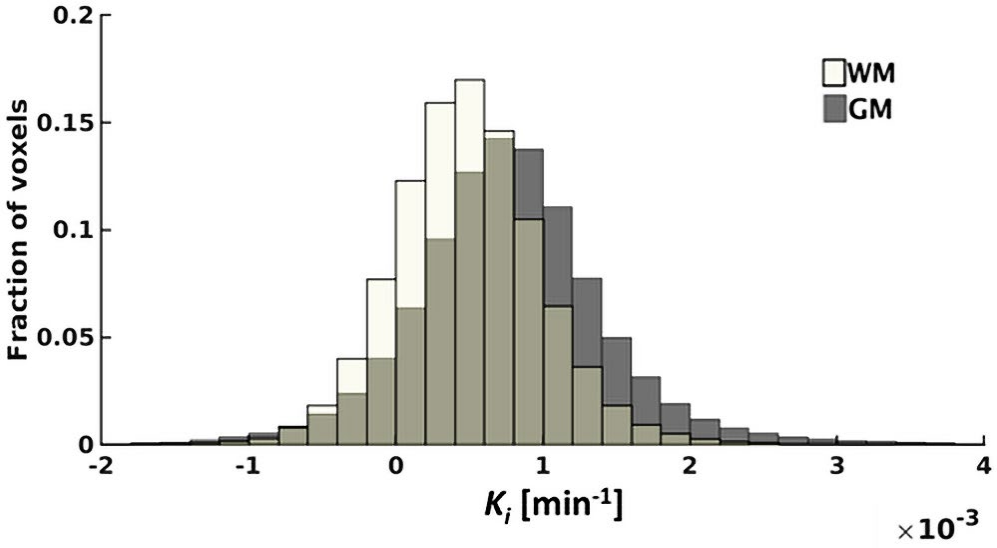
A representative example of the histogram acquired from 1 subject containing the *K_i_* values obtained in WM and GM

Representative concentration curves of venous blood, WM, and GM for 1 subject are depicted in Figure 5. A representative histogram of the *K_i_* values of WM and GM for 1 subject is shown in Figure 6. This figure illustrates the variation of voxel *K_i_* values, and shows a higher mean *K_i_* in GM compared with WM.

**FIGURE 7:**
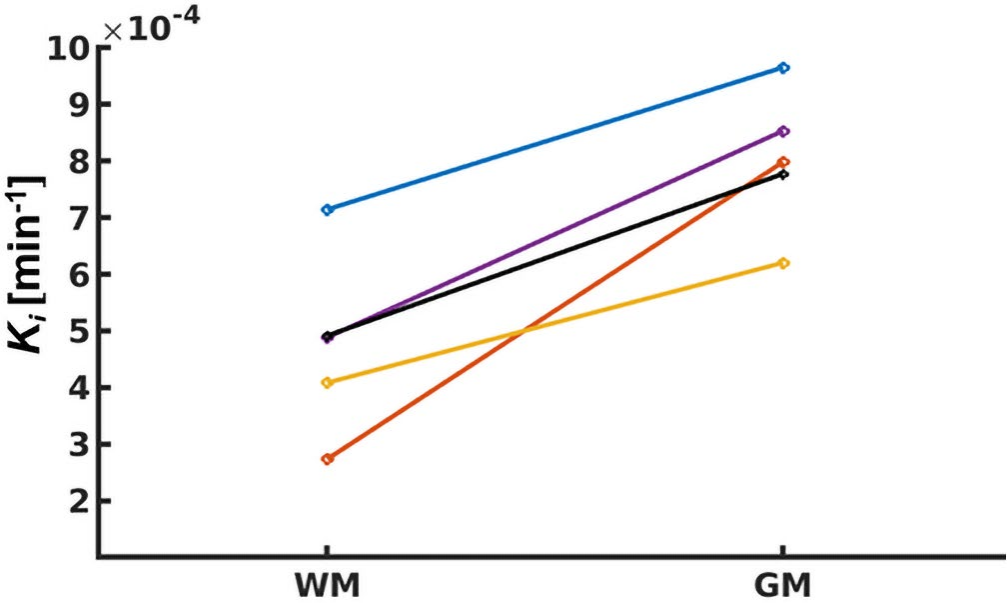
Pair‐wise plot of *K_i_* for WM and GM for each individual subject

The mean *K_i_* in WM over all subjects was 4.8 × 10^−4^ (SD 1.6 × 10^−4^) min^−1^, whereas the mean *K_i_* in GM was 8.0 × 10^−4^ (SD 1.3 × 10^−4^) min^−1^. Figure 7 shows the *K_i_* values of WM and GM for each subject. Significantly higher *K_i_* values were found in GM compared with WM (*p* = .04).

The mean *v_p_* in WM and GM was 3.4 × 10^−2^ (SD 0.5 × 10^−2^) and 4.9 × 10^−2^ (SD 0.4 × 10^−2^), respectively. The *v_p_* values also showed significantly higher values in GM compared with WM (*p* = .04).

## 4 DISCUSSION

In this study, we explored the feasibility of a sparsely time sampled DCE‐protocol using three T_1_ maps, for the detection and quantification of subtle BBB leakage at 7 T MRI. The conducted computer simulations showed that two postcontrast T_1_ maps with a most suitable time interval of approximately 20 minutes were able to detect the difference in subtle BBB leakage between normal WM and GM. Furthermore, the in vivo measurements demonstrated that the protocol had sufficient sensitivity to detect subtle leakage in normal‐appearing brain tissue.

The number of studies using DCE‐MRI to measure BBB integrity is increasing. Many studies complete DCE MRI examinations within several minutes (e.g., Chassidim et al^5^), which is sufficient to measure highly increased BBB permeability, occurring in cerebral diseases such as brain tumors and acute stroke.^3^, ^24^, ^25^, ^26^ However, for subtle BBB leakage, which has been linked to various types of cerebrovascular disorders (e.g. dementia, multiple sclerosis, cerebral small vessel disease), a long acquisition time is required, as was shown by simulations and in vivo measurements in previous studies.^4^, ^7^, ^18^, ^27^, ^28^ The need of a long acquisition time was supported by the findings of the simulations performed in the current study, which showed a better precision for later sample points.

Our study considered the long acquisition time required to detect subtle BBB leakage, and used the slow extravasation of CA to explore whether sparse time sampling would enable the detection of subtle leakage. Sparse sampling has the additional advantage of using the available scanning time more efficiently, as other relevant sequences can be scanned in between those few sample points. Because the CA strongly shortens the T_1_ relaxation time, the influence of CA on T_1_‐weighted scans should be evaluated before performing this type of sequences in the free time interval. T_2_‐weighted and $T_{2}^{*}$‐weighted images are less sensitive to the presence of gadolinium, and depending on the research question, can be performed with the presence of contrast agent. For example, Firat et al performed DWI 6‐10 minutes after contrast administration and reported no significant changes in the results.^9^ Contrast‐enhanced FLAIR can even facilitate the detection of lesions,^10^ and contrast‐enhanced SWI can be beneficial for visualizing deep veins^11^; however, it can be disadvantageous when focusing on the detection of microbleeds. Adding these sequences may make the BBB measurements more clinically attractive, as it is easier to incorporate these scans in the MRI protocol, without substantially increasing the total scan time.

The leakage measurements were performed by acquiring quantitative T_1_ maps. Previously, Taheri et al performed a continuous acquisition of a series of quantitative T_1_ maps with a relatively low temporal resolution of 3 minutes and 30 seconds, instead of the conventionally used dynamic T_1_‐weighted images with a high temporal resolution.^14^ Their study already recognized the advantage of directly converting the T_1_ values into CA concentrations, without the use of lookup tables or computational conversions, as is custom for T_1_‐weighted sequences used in conventional DCE‐MRI. Furthermore, T_1_ mapping has the advantage of being less influenced by interscanner inconsistencies, coil inhomogeneities, scanner drift, and acquisition parameter details, which have been reported to increase the noise for subtle BBB leakage.^14^, ^27^, ^29^, ^30^, ^31^ Because T_1_ mapping is more robust than T_1_‐weighted sequences, this might enable quantitative comparison of the results between different centers. Our proposed protocol combines these benefits with the advantages of an ultrahigh‐field 7 T MRI scanner, which allows the acquisition of high spatial‐resolution images in combination with a high SNR. The spatial resolution obtained in this study (i.e. 1.7 mm^3^) is higher compared with the typical spatial resolution in conventional DCE‐protocols (i.e. 2‐10 mm^3^).^4^

In this study, a relatively slow CA infusion rate was used, as pilot experiments using a clinically more common injection rate (3.0 mL/s) resulted in signal enhancement with a substantial $T_{2}^{*}$ signal decay for the fast perfusion scan. Such $T_{2}^{*}$ induced signal degradation results in underestimation of the CA concentration. Therefore, we chose to use a lower injection rate of 0.3 mL/s, which minimized any $T_{2}^{*}$ effects.

Computer simulations were conducted to assess the optimal sampling time points for the detection of subtle BBB leakage. As input for these simulations, we used leakage rates of WM and GM in normal brain tissue and leakage rates found in cerebrovascular disease. The values for the latter were based on the results reported by Montagne et al, which corresponded to an increase in *K_i_* of 20%. This range of values enabled investigating the effect of increasing leakage rates to find the most suitable time point.^16^

As a first objective of these simulations, the minimum number of sampling points after CA administration required for the detection of BBB leakage was determined by comparing the precision of *K_i_* for varying numbers of T_1_‐map acquisitions. Compared with the continuous scanning of T_1_ maps, the acquisition of the minimum of two postcontrast T_1_ maps resulted in a relatively small decrease in precision (−18%), while the bias was negligible (−0.5 %). It should be noted that a higher SD contributes to more variation in leakage rates between subjects. A lower precision, therefore, potentially leads to an increase in the required sample size to detect group‐level effects. However, this lower precision is still sufficient to detect subtle BBB leakage in WM and GM and the corresponding differences. Considering the trade‐off between obtaining the highest precision as possible for the quantification of subtle BBB leakage, and being able to scan other relevant sequences in between the (DCE) sampling points, we feel the increase in precision therefore does not outweigh the benefits of including additional sequences.

The simulations determining the optimal time interval between the two postcontrast T_1_ maps showed that it was beneficial to acquire the last T_1_ map as late as possible, to ensure the most precise leakage rates. This precision increase can be explained by the larger horizontal distance between the two data points in the Patlak plot, due to the longer time interval between the sampling time points. Assuming that the volume of distribution ratio C_t_(t)/C_p_(t) has the same error, this error will propagate into a larger uncertainty in the slope value (i.e. the *K_i_*) for a shorter time interval. Another advantage of a later time point is that the CA has more time to leak into the tissue, which results in an increased contrast‐to‐noise ratio.^32^ However, the simulations showed that when acquiring the T_1_ map at a later time point, the accuracy decreases (bias). This effect increased when the “true” *K_i_* was higher (e.g. in GM, aging, cerebrovascular disease). This decrease in accuracy can be explained as the Patlak approach ignores backflux of CA into the blood, which will result in an underestimation of *K_i_*. Because the bias was very small, we acquired the last T_1_ map as late as possible in this study. Most importantly, the simulations show that acquiring a late postcontrast T_1_ map is much more beneficial to improve the precision than acquiring multiple T_1_ maps (Figure 3 vs. Figure 4A,B).

The in vivo measurements show promising results for the protocol with sparse time sampling. Realistic concentration curves were obtained for blood, WM and GM, with measured leakage rates in the same order as reported in literature.^6^, ^14^, ^26^ Furthermore, the measurements were sensitive enough to detect significantly higher leakage rates and blood plasma volume fractions in GM compared with WM. These results for *v*_p_ are in accordance with histology and other in vivo imaging studies, which show a higher vascular volume and vessel surface area in GM compared with WM.^33^, ^34^, ^35^ The higher *K_i_* values found in this study can be explained by the larger vessel surface area in GM, as argued by Varatharaj et al.^36^ This study has some limitations. First, a small sample size was used in this study.

However, even given this small sample size, the potential of the sparsely sampled MRI protocol, especially the high sensitivity to low gadolinium concentrations in brain tissue, was shown. Second, due to the relatively long acquisition time of the T_1_ maps, subject motion was observed in a few subjects. This resulted in blurring and mixing of the signal intensities of WM and GM at the tissue transitions, despite the high spatial resolution obtained with 7 T. Furthermore, the precontrast and postcontrast T_1_ maps have different spatial resolutions, and therefore will be subject to different levels of partial volume effects. This error propagates to the *K_i_* maps when converting the MR signals to concentrations. When comparing the *K_i_* values of all subjects, these were all in agreement with biological expectations and literature, as discussed previously. Therefore, one could argue that the motion artifacts and partial volume effects mostly influence the local *K_i_* values and are averaged out when quantifying the *K_i_* of larger regions of interest. The effects of subject motion may be further reduced using shorter T_1_ mapping sequences. The T_1_ mapping sequence used in this study had an acquisition time of approximately 4 minutes, and yielded one data point. With emerging acceleration techniques, this acquisition time might be reduced to obtain a shorter scan time per time point with comparable spatial resolution and SNR.^37^ As an additional advantage, the shortening of acquisition time would expand the available time for other sequences even further.

## 5 CONCLUSIONS

This proof‐of‐principle study demonstrates that a sparsely time‐sampled DCE‐ MRI protocol, which consists of one precontrast, a dynamic perfusion scan, and two postcontrast T_1_ maps with a 20‐minute interval, is able to detect subtle BBB leakage in normal‐appearing brain tissue with sufficient accuracy and precision. The time efficiency of this protocol provides possibilities for future studies to incorporate BBB measurements more easily in clinical and research protocols. Moreover, acquiring temporally sampled quantitative T_1_ maps for leakage measurements, rather than continuous dynamic scanning T_1_‐weighted images, may reduce the influence of interscanner variability on the leakage‐rate measurements and should facilitate the comparison of data acquired in different centers.
